# Around the Hospitals

**Published:** 1897-07-10

**Authors:** 


### AROUND THE HOSPITALS.

[Notice to the Managers of Institutions.—Special space is now reserved for the insertion of notices of hospital meetings and festivals. The Editor requests that all notices may reach The Hospital Office, 28 & 29, Southampton Street, Strand, London, W.C., at least one week before the date of each meeting.]

**Hereford General Infirmary.**—At this infirmary, (which was established in 1776 on ground presented to it in perpetuity by Lord Oxford) 3,837 patients were treated during 1896, viz, 694 in-patients and 3,143 out-patients. The report issued each year is a most meagre one, consisting chiefly of the accounts, of which a fairly full statement is set out, and a brief record of the in and out-patients. Details, such as the average number of beds occupied, the average length of a patient's residence in the hospital, or any comparisons with the work of previous years are conspicuous by their absence.

**Birmingham General Hospital.**—Although not so pronounced as in the case of the Leeds General Infirmary (see page 239), the increase in the work done by the Birmingham General Hospital during the last thirty years has been enormous, as will be seen from the following:—

| | 1866. | 1876. | 1886. | 1896. |
|---|---|---|---|---|
| In-patients ... | 2,600 | 2,829 | 3,649 | 4,486 |
| Out-patients | 19,830 | 24,615 | 41,543 | 52,203 |
| Total ... | 22,430 | 27,444 | 45,192 | 56,689 |

Large as this increase is the work which will have to be done in the new buildings is likely to grow to even a greater extent. For this reason the slow growth of the annual subscription list—we might almost say the lack of growth in the subscription list—is a matter for serious consideration. In 1866 the subscription list amounted to £4,871; it has fluctuated between £4,500 and £5,500 in the years which follow, and in 1896 it stands at £5,144, less than £300 more than it was thirty years before. The result of the special appeal made for new annual subscriptions in November last does not, of course, to any extent affect these figures, but that this result is not as encouraging as it might be, is evidenced by the statement in the report of the committee, dated February 26th, 1897, that "the amount of the subscription list is alarmingly below the amount which will be required to maintain the hospital on the larger scale of work upon which it is about to enter." As we have mentioned above, 56,689 patients were treated during 1896, the total ordinary expenditure amounting to £17,884, an increase of about £1,200 on the two previous years. The average cost of each in-patient is worked out at £2 16s. 6d., an increase over the four previous years 1895, 1894, 1893, and 1892 of 5s. 5d., 2s. 8d., 5s. 7d., and 8s. 2d. per patient respectively: each out-patient the authorities estimate as costing 2s. The in-patients averaged 238 daily and remained in the hospital on an average 23 days, so that each cost about 17s. a week. The household averaged 129 and cost weekly about 7s. 2d. per head. The increase in the number of serious surgical operations performed in the hospital has continued, as is shown by the following figures: In 1892 649 operations were performed; in 1893, 752; in 1894, 854; in 1895, 1,055; and in 1896, 1,090. The accounts are made up on the uniform system, but we should like to see the £700 odd received from workmen placed under the heading provided for workmen's contributions in this form of accounts instead of being quried under the heading of "Annual subscriptions."

